# Community Voice Matters: Lessons from Field Studies of Malaria

**DOI:** 10.4269/ajtmh.20-0441

**Published:** 2020-09

**Authors:** Celina Aju-Ameh

**Affiliations:** Department of Zoology, University of Jos, Jos, Nigeria

I have always been curious about arthropods and the diseases they transmit to humans. Growing up in a malaria-endemic area (Benue, Nigeria) exposed my family members and me to the revulsions of malaria disease. Children were the worst hit. We had all sorts of symptoms (headaches, vomiting, joint pains, high fever, convulsion, loss of appetite, etc) and sometimes death. Chief among my symptoms was convulsions. The story goes that my malaria episodes and convulsions were so frequent that I literally formed a song that says *I have my neck on a tree singing will I die or will I survive*? Somehow, I survived but not without an uncountable number of malaria episodes with chloroquine injections and the adverse itching side effects.

I became interested in mosquito bionomics, aspiring to study them and get to the bottom of the illness that prevented us from going to school and playing with our friends. Aspiration became a reality when for my master’s degree project I worked on *Aedes* (Stegomyia) *aegypti* and more recently for my PhD, I took on *Anopheles gambiae*—our resident enemy. Starting with the study community’s commonest illness—“Iba Maria” (fever-malaria by the locals), I set out on full time research.

In the course of the project, I had to pick up indoor biting and resting mosquitoes between the hours of 06:00 and 09:00 over a period of 2 years (2015–2016), four times in a year. Prior advocacy visits prepared the occupants for the early morning exercise. The occupants would step out on our arrival; we would move in and cover the furniture and floor with white sheets. Next, we shut the windows and doors and sprayed the room with insecticides. Then, we stepped out for a bit and reentered after about 10 minutes to pick up knocked down mosquitoes on the white sheets.

The knocked down insects were readily visible on the white sheet but not all the communities accepted the white sheets, the reason being that it is generally used for burial ceremonies—more specifically to overlay corpses. So, we bought and replaced the white sheets with golden brown/yellowish sheets. With these new sheets, we were warmly welcomed and made good progress in the first one and half years of the project.

Way into the second year, we headed out to the study communities for the last field visit and met head on another challenge. The story about the death of a family from the use of adulterated spray had made the rounds and set the stage for resistance from affected communities. The seller in the local market from whom we bought the sprays had confirmed the news and suggested three brands that may still be acceptable. Although the project brand was one of the three brands suggested, the affected communities were unwilling to let us spray their sleeping places. We needed time for this community to trust us again. Therefore, we continued our project with nearby communities that were unaffected and used that to win back the trust of the affected communities. The project participants in the affected communities were as apprehensive as we were. My fears were predicated on two trains of thought: what if there were underlying issues or other confounders besides the spray that could have been responsible for the unproven deaths; and, should anything go wrong, who takes responsibility?

Nothing prepared me for what was coming next. As we moved from community to community, I discovered some families had no nets, whereas others had surplus, with some intact in their packs unused. Without planning, I started to ask questions about redistributing the nets; I wanted to recover the unused nets and pass them on to those who didn’t have any. Mosquito bed nets provide both personal and community protection. My unplanned questions received spontaneous answers. Whereas some were diligent with daily use, others abhorred the nets. Families reported that they were having adverse reactions such as skin irritation or red and teary eyes from the insecticide-treated bed nets. I was truly shocked when they told me they thought that these nets kill newborn babies!

On getting back, I went through the semistructured questionnaires used to elicit information on malaria-related knowledge, attitudes, and practices during the cross-sectional survey. Among the reasons recovered from the survey questionnaires for nonuse of these nets or preference for nontreated bed nets was the adverse effect of the treated bed nets. One respondent actually wrote that long-lasting insecticide-treated nets (LLINs) almost suffocated her to death. Two respondents with secondary-level education in the urban community vowed never to use the LLINs because it is lethal. Long-lasting insecticide-treated nets are one of the World Health Organization’s core control tools and a major intervention strategy driving the national elimination goal. As a researcher and a mother whose daughters battled malaria over the years, a barrage of questions poured through my mind. Did the bereaved mothers air the nets, following through on the usage instructions? What brand of insecticide was used to treat the nets in question? Were the nets obtained from the approved distribution channels (antenatal/immunization clinics) or the open market? How do we enhance the integrity of LLINs among these people? Could this be a contributing factor to the high degree of human–vector contact established in the study? The deceased babies’ voices are silent forever, but their mothers’ voices continue to speak for the pediatric population in that locality. The mothers echoed the gnawing pain of loss spreading their theory, albeit with no evidence, while sharing a real and an irreparable loss. Personally, my daughters would break out with skin irritations—redness, swelling on the face, and teary eyes. To avoid or reduce the severity of the discomfort, we would air the nets for 48–72 hours and sometimes more before first use.

One more challenge, and this left me with a key lesson learnt: It is important to be accessible, to engage, and to actively listen to the people in the study communities beyond the first advocacy visit. I was so engrossed with my indoor mosquitoes that I was too blind to see mosquito nets hung outdoors. Fixated on my project objectives, I ignored complaints about outdoor bites until two forthright mothers stopped me right in my tracks and lashed out at me. I was told that I was always coming to pick indoor mosquitoes but that outdoor biting mosquitoes were much more troublesome at the time. It was a case of my perceived need versus their felt need. On sharing this experience with a senior member of the Ministry of Health at the local government level, she advised that I observe more closely nets hung on trees/sticks outdoors and revisit the study communities between 6:00 and about 10:00 pm. Of course, I had seen several, but I thought well, they could be airing the nets just like I do or just a few cases of unorthodox use. She said outdoor bites were now a huge challenge which had led to outdoor use of mosquito bed nets for evening sit-outs!

